# SGLT2 Inhibitors May Restore Endothelial Barrier Interrupted by 25-Hydroxycholesterol

**DOI:** 10.3390/molecules28031112

**Published:** 2023-01-22

**Authors:** Agnieszka Pawlos, Marlena Broncel, Ewelina Woźniak, Łukasz Markiewicz, Agnieszka Piastowska-Ciesielska, Paulina Gorzelak-Pabiś

**Affiliations:** 1 Laboratory of Tissue Immunopharmacology, Department of Internal Diseases and Clinical Pharmacology, Medical University of Lodz, 91-347 Lodz, Poland; 2 BRaIn Laboratories, Medical University of Lodz, 92-216 Lodz, Poland

**Keywords:** SGLT2i, endothelial integrity, endothelial barrier, VE-cadherin, empagliflozin, canagliflozin, dapagliflozin, atherosclerosis, 25-hydroxycholesterol

## Abstract

SGLT2 (Sodium-glucose Cotransporter-2) inhibitors are newer glucose-lowering drugs with many cardiovascular benefits that are not fully understood yet. Endothelial integrity plays a key role in cardiovascular homeostasis. 25-hydroxycholesterol (25-OHC), which is a proatherogenic stimuli that impairs endothelial barrier functions. VE-cadherin is an endothelial-specific protein crucial in maintaining endothelial integrity. The aim of this study was to assess the influence of SGLT2i on the integrity of endothelial cells interrupted by 25-OHC. We also aimed to evaluate whether this effect is associated with changes in the levels of VE-cadherin. We pre-incubated HUVECs with 10 μg/mL of 25-hydroxycholesterol (25-OHC) for 4 h and then removed it and incubated endothelial cells with 1 μM of empagliflozin, 1 μM canagliflozin, or 1 μM dapagliflozin for 24 h. The control group included HUVECs cultured with the medium or with 25-OHC 10 μg/mL. The integrity of endothelial cells was measured by the RTCA-DP xCELLigence system, and VE-cadherin was assessed in confocal microscopy. Our results show that SGLT2 inhibitors significantly increase endothelial integrity in comparison to medium controls, and they improve endothelial cell integrity interrupted by 25-OHC. This effect is associated with significant improvements in VE-cadherin levels. SGLT2i: empagliflozin, canagliflozin, and dapagliflozin have a beneficial effect on the endothelial cell integrity and VE-cadherin levels reduced by 25-OHC.

## 1. Introduction

According to the recent results from the Global Burden of Diseases, Injuries, and Risk Factors Study (GBD) in 2019, cardiovascular diseases (CVD) were reported as responsible for one third of deaths globally, and atherosclerosis is regarded as the pathophysiological target for most CVD worldwide [1]. Flozins are newer glucose-lowering agents that block glucose reabsorption from the urine by inhibiting SGLT2 receptors in the proximal kidney tubules [2]. The results of EMPA-REG outcome trial (The Empagliflozin Cardiovascular Outcome Event Trial in Type 2 Diabetes Mellitus Patients–Removing Excess Glucose (EMPA-REG) OUTCOME) have shown that for patients taking empagliflozin, the risk of death from cardiovascular causes was reduced by as much as 38%, and the risk of death from any cause reduced by 32% [3]. Thanks to SGLT2 inhibitors and their pleiotropic effects, the current approach to treating T2DM is aimed not only at reducing glucose levels, but also improving life expectancy by reducing cardiovascular risk. Recently, flozins have also shown benefits in patients without diabetes, in heart failure and in chronic kidney disease [4]. According to the recent EMPEROR-Preserved study, empagliflozin is the only drug that improves life expectancy in patients with heart failure with preserved ejection fraction (HFpEF) [5,6]. The exact mechanism of flozins beneficials is not fully elucidated. We hypothesize, that the possible mechanisms of reducing cardiovascular mortality include beneficial effects, which is mediated by a direct influence on endothelial cells. Endothelial cells are key players in the development of atherosclerosis at different stages of the disease [7]. The loss of endothelial integrity occurs at the initial part of atherogenesis, and it leads to increased permeability to proatherogenic and proinflammatory stimuli, which then built-up atherosclerotic plaque [8]. 25-hydroxycholesterol (25-OHC) is an oxidized form of cholesterol that takes part in the build-up of atherosclerotic plaques [9]. In our previous studies, we have observed that 25-OHC impairs endothelial integrity and leads to increased permeability [10]. The integrity of the endothelial layer is maintained by proteins, which form tight and adherens inter-cellular junctions. VE-cadherin (vascular endothelial cadherin), which is also known as cadherin 5 and CD144, is tissue-specific, and its expression is limited only to endothelial cells. In other cell types its promoter repressed [11]. Moreover, VE-cadherin is the most important protein in maintaining the endothelial barrier architecture, as only its knockdown among other cell–cell adhesion molecules is lethal in rats [12].

The aim of this study was to investigate whether SGLT2 inhibitors affect the integrity of endothelial cells and if they are able to restore HUVECs integrity interrupted by 25-hydroxycholesterol. We also aimed to evaluate whether this effect is associated with changes in the levels of VE-cadherin.

## 2. Results

In our research model, first we incubated HUVECs with empagliflozin 1 μM, canagliflozin 1 μM, or dapagliflozin 1 μM for 24 h, and evaluated their influence on the integrity of endothelial cells in the RTCA-DP system and on the VE-cadherin levels in confocal microscopy in comparison to the medium control. Subsequently, we aimed to evaluate if SGLT2is are able to improve the integrity of endothelial cells and the VE-cadherin levels decreased by 25-OHC. We pre-incubated HUVECs with 10 μg/mL of 25-hydroxycholesterol (25-OHC) for 4 h and then removed it and incubated endothelial cells with 1 μM of empagliflozin, 1 μM canagliflozin, or 1 μM dapagliflozin for 24 h. The control group included HUVECs cultured with the medium or with 25-OHC 10 μg/mL.

### 2.1. SGLT2 Inhibitors: Empagliflozin, Canagliflozin, and Dapagliflozin Significantly Increase Endothelial Integrity in Comparison to the Medium Control

**Figure 1:**
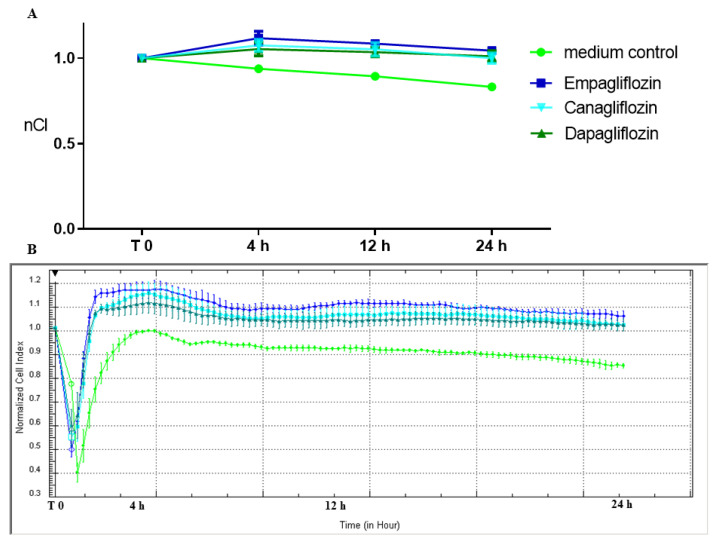
(**A**) The effect of SGLT2 inhibitors empagliflozin (1 µM), canagliflozin (1 µM), and dapagliflozin (1 µM) on the integrity of HUVECs. Data were collected by real-time cell electric impedance sensing system. (**B**) Representative plot from the real-time cell electric impedance sensing system, each line represents the mean of three wells of each condition.

The integrity of HUVECs incubated with all SGLT2is was significantly higher that with the medium control in all three time points: 4 h, 12 h, and 24 h (Table 1), (Figure 1A,B). After 24 h of stimulation, the integrity of HUVECs incubated with empagliflozin, canagliflozin, or dapagliflozin was, respectively, 20.2%, 17.0%, and 17.1% higher than the medium control (Table 1). There were no significant differences between the integrity of endothelial cells incubated with empagliflozin vs. canagliflozin vs. dapagliflozin (Table 1).

### 2.2. SGLT2 Inhibitors: Empagliflozin, Canagliflozin, and Dapagliflozin Improved Endothelial Cell Integrity Interrupted by 25-OHC

**Figure 2:**
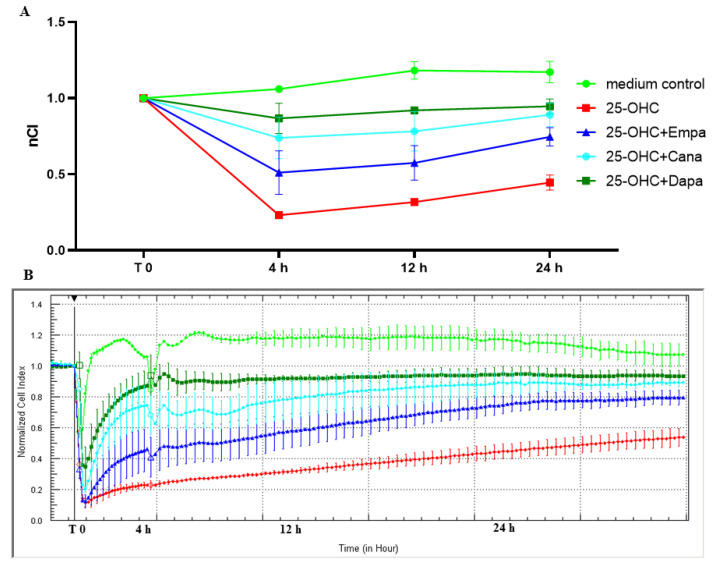
(**A**) The effect of SGLT2 inhibitors empagliflozin (1 µM), canagliflozin (1 µM), and dapagliflozin (1 µM) on the integrity of HUVECs that are pre-stimulated with 25-OHC for 4 h. Data collected by real-time cell electric impedance sensing system. (**B**) Representative plot from the real-time cell electric impedance sensing system, each line represents the mean of three wells of each condition.

To evaluate if SGLT2 inhibitors exert a beneficial effect against proatherogenic stimuli on the barrier function of endothelial cells, we have pre-incubated HUVECs for 4 h with 25-OHC and then removed it and incubated HUVECs for 24 h with SGLT2is. 25-hydroxycholesterol significantly reduced the integrity of HUVECs by 78.3%, 72.9%, and 61.54%, after 4, 12, and 24 h, respectively (Table 2, Figure 2). This effect could not be caused by the reduction of the endothelial cells’ viability, as we have described in the methods section, none of the tested substances influenced HUVECs viability (Table 3). All tested flozins at each time point significantly improved the integrity of the endothelial cells that were interrupted by 25-OHC. In the 24 h timepoint, empagliflozin, canagliflozin, and dapagliflozin significantly improved the endothelial cell integrity by 66.7%, 97.8%, and 111%, respectively, in comparison to 25-OHC (Table 2).

### 2.3. SGLT2 Inhibitors: Empagliflozin, Canagliflozin, and Dapagliflozin Completely Rescue VE-Cadherin Levels Decreased by 25-Hydroxycholesterol

**Figure 3:**
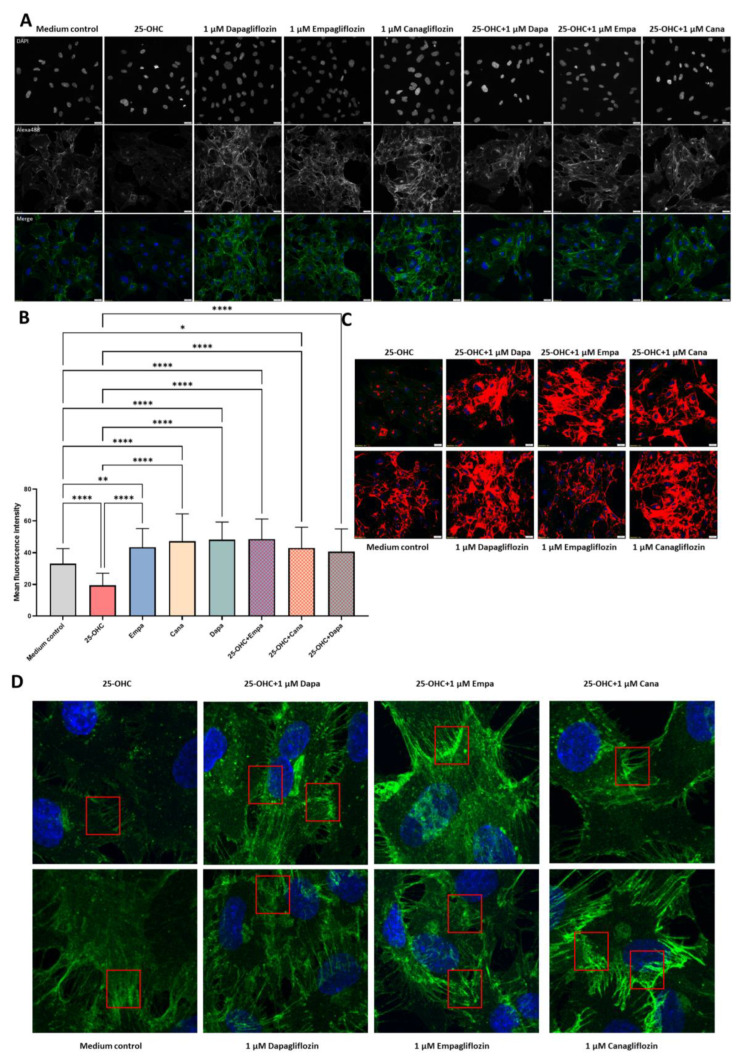
The expression and distribution of VE-cadherin in endothelial cells after treatment with 25-OHC and selected flozins. HUVEC cells were treated with medium (control), 10 μg/mL 25-OHC (4 h), followed by 24 h incubation with or without 1 μM dapagliflozin, empagliflozin, or canagliflozin. (**A**) Cells were stained for VE-cadherin (green) and DAPI (blue). (**B**) Quantification of VE-cadherin protein levels measured as mean fluorescence intensity in cell membrane (*n* = 30–40 cells). (**C**) Visualization by color mask (red) of all fluorescence points with intensity ≥1000. (**D**) The selected enlargements of a mature cell–cell junction (red frames). White scale bars represent 20 μm. Data are presented as mean ± SD. * *p* < 0.05, ** *p* < 0.01, and **** *p* < 0.0001.

To further explore the changes in the endothelial layer integrity revealed by the RTCA-DP system, we used immunofluorescence imaging. We tested if the effect of treatment with selected flozins on the cell–cell junctions may be related to the expression levels and/or the distribution of VE-cadherin in HUVEC cells. All of the tested flozins (1 µM empagliflozin, 1 µM canagliflozin, and 1 µM dapagliflozin) significantly elevated the VE-cadherin expression levels calculated as the mean fluorescence in the cell (Figure 3A,B). Moreover, the 25-hydroxyholesterol-dependent VE-cadherin decrease was completely rescued by the incubation with tested drugs (Figure 3C). This effect was even more profound when we assessed the VE-cadherin concentration only in the vicinity of the cell membrane (Figure 3D).

## 3. Discussion

SGLT2 inhibitors bring many benefits to patients with cardiovascular diseases, regardless of their glycemic status, and the exact mechanism of this phenomenon is yet to be elucidated. This study investigates the direct effect of the SGLT2 inhibitors on the integrity of endothelial cells, which may be one of the possible mechanisms of their beneficial pleiotropic effects in cardiovascular diseases. The integrity of the endothelial monolayer was measured using bioimpedance in the RTCA-DP system. The measurement of the endothelial cells’ integrity was followed by the assessment of VE-cadherin, a highly endothelial-specific protein crucial in AJs (adherens junctions), which was quantified using immunofluorescence imaging.

Surprisingly, according to our results the integrity of HUVECs exposed to SGLT2i was significantly higher than the control with the medium (Table 1, Figure 1). This effect was accompanied by a significant enrichment of VE-cadherin in the endothelial membrane area, where the formation of AJs occurs (Figure 3D). Such results may reflect the high potential of empagliflozin, canagliflozin, and dapagliflozin to improve the endothelial barrier function, which may be one of the possible mechanisms of their clinical benefits. The improvement of the endothelial barrier function caused by flozins may be due to their ability to modulate cellular metabolism, observed even in cells that do not express SGLT2 receptors [13]. This effect is called the ‘state of fasting mimicry’, because SGTL2 inhibitors cause transcriptional changes similar to those occurring in cells responding to starvation, including the activation of SIRT1/AMPK [14]. Interestingly, an in silico analysis of the SGLT connection network revealed the shortest connection path between SGLT2 and SIRT1 [15]. According to the results obtained by Zhang W. et al., the activation of SIRT-1 was associated with improved endothelial barrier functions and higher membrane levels of VE-cadherin [16,17]. Moreover, stimulation of SIRT1 leads to the activation of the hypoxia-inducible factor-2α (HIF-2α), which significantly improves the endothelial cells’ barrier functions [18,19]. That phenomenon can be explained by the increased expression of VE-cadherin caused by HIF-2α, which irrespectively of hypoxia activates VE-cadherin gene expression via specifically activating its promoter, by binding with its HRE region [20]. Our in vitro findings seem to have clinical relevance, as according to a recent meta-analysis, SGLT2is significantly improve FMD (flow mediated dilation), which reflects endothelial function [21].

After obtaining the results of the direct beneficial effect of SGLT2 inhibitors themselves on HUVECs integrity and the level of VE-cadherin, we have further explored their ability to revert the damaging effect of 25-hydroxycholesterol (25-OHC), and thus to possibly affect atherogenesis. In our ‘in vitro model of atherosclerosis’ first, we have pre-incubated HUVECs with 25-OHC, which caused a significant impairment of the endothelial cells’ integrity and the decrease of VE-cadherin levels (Figure 2). Then, we removed 25-OHC and incubated HUVECs with empagliflozin, canagliflozin, or dapagliflozin for 24 h. We have shown for the first time that empagliflozin, canagliflozin, and dapagliflozin counteracted the endothelial barrier disruption and restored VE-cadherin levels that were decreased by 25-OHC (Figure 2 and Figure 3). The deterioration of the endothelial cells’ integrity exposed to 25-OHC could not have been caused by the decrease in cellular viability, because neither 25-hydroxycholesterol nor empagliflozin, canagliflozin, or dapagliflozin influenced the endothelial viability (Table 3). 25-OHC, which is an oxidized form of cholesterol, acts as a proatherogenic stimuli and exerts a variety of effects in endothelial cells, including promoting inflammation, increasing oxidative stress, and affecting endothelial barrier integrity [10,22]. The interruption of endothelial integrity in response to proatherogenic stimuli is a crucial moment in atherogenesis, and VE-cadherin is the leading protein maintaining endothelial integrity [8,23]. The molecular basis of VE-cadherin-dependent endothelial cells’ integrity has been under intense investigation, leading to the identification of key proteins: catenins. The role of the β-isoform catenin in maintaining endothelial barrier integrity that is dependent on VE-cadherin is crucial, as it links the VE-cadherin junction complex with cytoskeleton and thus, promotes cell–cell adhesion. It also participates in the Wnt signaling pathway [24]. In our study, 25-OHC decreased the VE-cadherin levels and endothelial integrity, and one of the possible mechanisms of that phenomenon might be Wnt/β-catenin signaling, which is also reduced by 25-OHC, according to recent evidence [25]. Moreover, in line with the results obtained by Cai C. et al., empagliflozin was able to activate β-catenin by preventing its phosphorylation, which may also explain our results to some extent [26]. Another possible explanation underlying our results may be the anti-oxidative effect of SGLT2is. As we have shown in our previous studies, 25-OHC induces oxidative stress in HUVECs. The production of ROS (reactive oxygen species) measured by DCF fluorescence intensity was significantly higher in HUVECs incubated with 25-OHC in comparison to the medium control, which was associated with DNA oxidative damage of purines and pyrimidines [22]. Moreover, according to our results, empagliflozin indirectly promoted DNA repair by reducing the production of ROS [27]. Increased ROS production contributes to the phosphorylation of VE-cadherin, which leads to its internalization and thus, the interruption of adhesive junctions, which leads to impaired endothelial barrier integrity [28]. According to results obtained by Li et al., empagliflozin, canagliflozin, and dapagliflozin ameliorated the endothelial barrier dysfunction and prevented VE-cadherin loss by the inhibition of ROS in HCAECs under cyclic stretch [29]. In contrary to our data, Uthman et al. reported that empagliflozin was not able to reverse the damaging effects of TNFα on HUVECs permeability [30]. This discrepancy can be attributed to different experimental designs. The authors pretreated cells with SGLT2is, while in our research model, the damaging agent was administrated before SGLT2is incubation. Another cause of different results may be different damaging factors. It may be possible that SGLT2is are able to reverse the endothelial damage inflicted by 25-OHC but not by TNFα in HUVECs; however, further studies are needed.

In our results, there was an interesting inconsistency; VE-cadherin levels were completely rescued by SGLT2 inhibitors, meanwhile the integrity of HUVECs was significantly improved, but still reduced in comparison to the medium control. We hypothesize that this phenomenon might be due to the fact that the rescue of the VE-cadherin levels might not be simultaneous with the rescue of VE-cadherin functionality. The level of the VE-cadherin level does not equal VE-cadherin functionality, which may explain the gap between fully rescued VE-cadherin levels and not-fully restored endothelial cells’ integrity in the xCELLigence system. The functionality of VE-cadherin is regulated by its phosphorylation; however, this mechanism is complex and the existing evidence is controversial [31]. According to Wessel et al., VE-cadherin is constitutively phosphorylated at Tyr731 in vivo, and dephosphorylation at that site affects leukocyte migration. Meanwhile, the phosphorylation of VE-cadherin at Tyr685 increases vascular permeability [32]. In light of conflicting information regarding little to no expression of SGLT2 in HUVEC cells, our data may suggest several mechanisms of SGLT2is-dependent regulations of VE-cadherin to be addressed in future research [33]. Those include modulating VE-cadherin activity through phosphorylation and controlling the amount of VE-cadherin available for an engagement at adherens junctions.

## 4. Materials and Methods

### 4.1. Chemicals

Trypsin with EDTA, trypsin neutralizing solution, endothelial cell growth medium-2 (EGM-2) with hydrocortisone, hFGF-B, VEGF, R3-IGF-1, ascorbic acid, hEGF, GA-1000, heparin, and fetal bovine serum (FBS), were purchased in Lonza (Basel, Switzerland). Empagliflozin (11575), dapagliflozin (11575), and canagliflozin (11575) were purchased from Cayman Chemical (Ann Arbor, Michigan, USA) and 25-OHC was purchased from Sigma-Aldrich (St. Louis, MO, USA). The antibodies used for immunofluorescence: VE-Cadherin (D87F2) XP^®^ Rabbit mAb #2500 were purchased from Cell Signaling (Warszawa, Poland), and anti-Rabbit (H+L) Alexa Fluor Plus 488 was purchased from Invitrogen (Waltham, MA, USA).

### 4.2. Cells

Human umbilical vein endothelial cells (HUVECs) (Lonza C2517A) were expanded in endothelial basal medium-2 (EGM-2) (Lonza, Clonetics, CC-3162) containing 10% fetal bovine serum (FBS), hydrocortisone, hFGF-B, vascular endothelial growth factor (VEGF), R3-IGF-1, ascorbic acid, hEGF, GA-1000, heparin, penicillin (100 U/mL), and streptomycin, (100 µg/mL) at 37 °C, 5% CO_2_. After reaching 80–90% confluence, the HUVECs were removed by treatment with 0.05% trypsin with 0.02% EDTA for three minutes and then neutralized by trypsin neutralizing solution. The viability of the cells was over 98% (Table 3). The trypan blue dye exclusion test was used to determine the number of viable cells. Data are expressed as mean ± SD.

### 4.3. Cell Treatment

**Figure 4:**
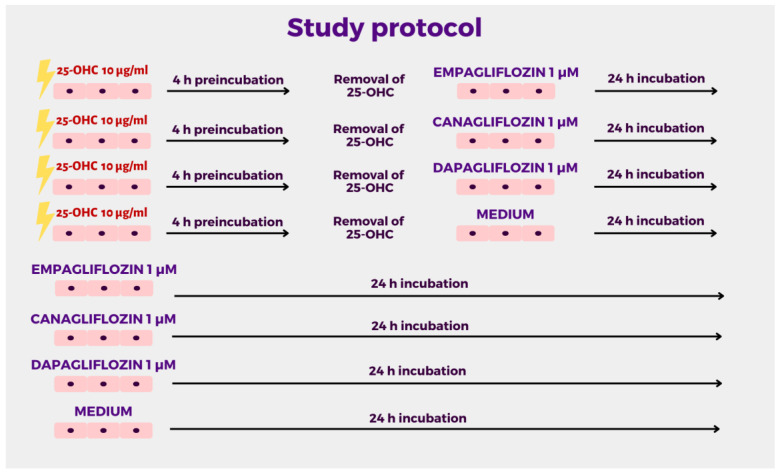
Study protocol.

Both trypsinized HUVECs were separately seeded on 24-well plates at a density of 100,000 cells per well in 600 µL proper medium. After reaching 80–90% confluence, HUVECs were stimulated with 25-hydroxycholesterol (10 µg/mL) for four hours. After 4 h of preincubation, 25-OHC was removed and HUVECs were incubated with empagliflozin 1 µM, dapagliflozin 1 µM, canagliflozin 1 µM, or medium for 24 h (Figure 4). After incubation, the compounds were discarded, and the cells were resuspended in EGM-2 medium. The concentrations of empagliflozin, dapagliflozin, and canagliflozin were selected based on pilot experiments and previous studies [34].

### 4.4. Cell Culture in the Real-Time Cell Electric Impedance Sensing System (RTCA-DP, xCELLigence)

The RTCA-DP xCELLigence system (Roche Applied Science ACEA Biosciences, Inc. 6779 Mesa Ridge Road Ste. 100, San Diego, CA, USA) allows cell growth status to be monitored in real-time on microelectrode-coated plates by tracking the electrical impedance signals. The impedance readout is expressed in arbitrary units, such as cell index (CI). The values reflect changes in the barrier properties, monolayer permeability, cell number, viability, adhesion, and morphology. The normalized cell index (nCI) is calculated by dividing CI at the normalized time by the original CI value. The rate of cell growth was determined by calculating the slope of the line between two given time points. The integrated software allows data to be collected each minute for any period of time.

In the present study, the impedance measurement system was used for dynamic and qualitative analysis of HUVEC cells. The trypsinized HUVEC cells were separately seeded on E-16 plates at a density of 10,000 cells per well in proper media, and any changes in CI were observed. After reaching the plateau phase, the cells were pre-incubated with 25-hydroxycholesterol 10 µg/mL for four hours, and then incubated with empagliflozin, dapagliflozin, and canagliflozin for 24 h. Cells were cultured with medium, and 10 µg/mL 25-hydroxycholesterol were used as controls.

Six wells were used for each file. Data for cell adherence were normalized (nCl) after four hours of preincubation by 25-hydroxycholesterol and before stimulation with empagliflozin, dapagliflozin, and canagliflozin. The HUVEC cultures were analyzed in the 4th, 12th, and 24th hours after induction, as the most significant changes could be seen at these time points.

### 4.5. Immunofluorescence and Confocal Microscopy

HUVEC cells were cultured on glass coverslips in a 24-well plate. Cells were fixed in 4% (*w*/*v*) paraformaldehyde in PBS for 10 min at room temperature (RT), and were washed in PBS (3 × 5 min). Fixed cells were incubated with permeabilization buffer (0.3% Triton X-100 in PBS) for 15 min at RT. Then, cells were washed in PBS (3 × 5 min) and blocked in blocking buffer (1% (*v*/*v*) with normal donkey serum, 10 mg/mL (*w*/*v*), bovine serum albumin, 0.1% (*v*/*v*), and Triton X-100 in PBS) for 1 h at RT. Primary antibodies were diluted in blocking buffer and used to stain cells overnight at 4 °C. After washing (3 × 5 min) in PBS containing 0.1% (*v*/*v*) Triton X-100, secondary antibodies were added in blocking buffer and incubated for 1 h at RT. The cells were washed as above and mounted with DAPI-containing antifade medium (EverBrite, Biotum). Microscopy was performed on an inverted Olympus IXplore SpinSR10 super resolution microscope system equipped with a 60 × oil objective and the Hamamatsu ORCA-Fusion cameras. All fluorescent images were corrected for background and negative controls. For the quantitative analysis of fluorescence intensities, all images were obtained with identical gain, offset, and laser power settings. Cells were manually annotated using an ImageJ and fluorescent intensities were measured with the help of an ImageJ software.

The following antibodies were used for immunofluorescence: VE-Cadherin (D87F2) XP^®^ Rabbit mAb #2500 (Cell Signaling) and anti-Rabbit (H+L) Alexa Fluor Plus 488 (Invitrogen).

### 4.6. Statistical Analysis

The distribution of particular variables was verified by the Shapiro–Wilk W-test, and homogeneity of variance was determined with the Brown–Fisher test. Tukey’s test was used as a post hoc test. A *p*-value < 0.05 was considered to be statistically significant.

Each analysis was performed in four independent experiments, with each experiment repeated twice or three times depending on the method. Statistical analyses were performed with the GraphPad Prism 9.0 (GraphPad Software, San Diego, CA, USA) and Statistica software (StatSoft, Inc., Kraków, Poland).

## 5. Conclusions

Our data showed that SGLT2 inhibitors (SGLT2is), regardless of substance (empagliflozin, canagliflozin, and dapagliflozin), can positively modulate the endothelial monolayer integrity by stimulating VE-cadherin expression. Moreover, the 25-hydroxyholesterol-dependent VE-cadherin decrease was completely rescued by the incubation with the tested flozins.

## 6. Limitations

Our study has several limitations that can be improved in further research:

●● Lack of VE-cadherin mRNA analysis;

●● No analysis of inflammatory response;

●● Lack of evaluation of the dependance of observed effects on SGLT2 receptors.

## Figures and Tables

**Table 1:** Summary of the normalized cell index (nCl) of HUVECs incubated with SGLT2 inhibitors empagliflozin (1 µM), canagliflozin (1 µM), and dapagliflozin (1 µM) in three time points T0, 4 h, 12 h, and 24 h. * Adjusted *p* value of Tukey multiple comparisons tests in 2-way ANOVA.

	nCI	T:0	T:4 h	T:12 h	T:24 h
mean ± SD	medium control	1 *±* 0	0.94 *±* 0.01	0.89 *±* 0.01	0.83 *±* 0.01
	Empa 1 µM	1 *±* 0	1.12 *±* 0.04	1.08 *±* 0.01	1.04 *±* 0.01
	Cana 1 µM	1 *±* 0	1.07 *±* 0.04	1.05 *±* 0.04	1.00 *±* 0.03
	Dapa 1 µM	1 *±* 0	1.05 *±* 0.04	1.03 *±* 0.03	1.01 *±* 0.03
Adjusted *p* value *	sp vs. Empa	>0.9999	<0.0001	<0.0001	<0.0001
	sp vs. Dapa	>0.9999	<0.0001	<0.0001	<0.0001
	sp vs. Cana	>0.9999	<0.0001	<0.0001	<0.0001
	Empa vs. Dapa	>0.9999	0.0133	0.0698	0.3398
	Empa vs. Cana	>0.9999	0.1580	0.3407	0.1356
	Dapa vs. Cana	>0.9999	0.6874	0.8257	0.9502

**Table 2:** Summary of the normalized cell index (nCl) of HUVECs that are pre-stimulated with 25-OHC for 4 h and then with SGLT2 inhibitors empagliflozin (1 µM), canagliflozin (1 µM), or dapagliflozin (1 µM), in three time points T0, 4 h, 12 h, and 24 h. * Adjusted *p* value of Tukey multiple comparisons tests in 2-way ANOVA.

	nCI	T:0	T:4 h	T:12 h	T:24 h
mean ± SD	Medium control	1 *±* 0	1.06 *±* 0.01	1.18 *±* 0.06	1.17 *±* 0.07
	25-OHC	1 *±* 0	0.23 *±* 0.02	0.32 *±* 0.02	0.45 *±* 0.05
	25-OHC+Empa 1	1 *±* 0	0.51 *±* 0.14	0.57 *±* 0.11	0.75 *±* 0.06
	25-OHC+Cana 1	1 *±* 0	0.74 *±* 0.14	0.78 *±* 0.13	0.89 *±* 0.08
	25-OHC+Dapa 1	1 *±* 0	0.87 *±* 0.1	0.92 *±* 0.02	0.95 *±* 0.05
Adjusted *p* value *	Medium control vs. 25-OHC	>0.9999	<0.0001	<0.0001	<0.0001
	Medium control vs. 25-OHC + Empa 1	>0.9999	<0.0007	<0.0001	<0.0001
	25-OHC vs. 25-OHC + Empa 1	>0.9999	0.0020	0.0019	0.0005
	Medium control vs. 25-OHC + Cana 1	>0.9999	<0.0009	<0.0001	0.0028
	25-OHC vs. 25-OHC + Cana 1	>0.9999	<0.0001	<0.0001	<0.0001
	Medium control vs. 25-OHC + Dapa 1	>0.9999	0.0007	<0.0001	0.0001
	25-OHC vs. 25-OHC + Dapa 1	>0.9999	<0.0001	<0.0001	<0.0001

**Table 3:** The level in the viability level of human HUVECs was determined by the trypan blue dye exclusion test. HUVECs were induced by 25-hydroxycholesterol (10 µg/mL), empagliflozin, dapagliflozin, and canagliflozin (1 µM). Mean ± SD was calculated from nine individual experiments. Statistical analysis was conducted using one-way ANOVA and post hoc Tukey’s test.

Compounds	Concentration	Cell Viability [%]	ANOVA I
Control	0 µg/mL	98.9 ± 0.8	-
25-hydroxycholesterol	10 µg/mL	97.3 ± 1.6	*p* > 0.05
Empagliflozin	1 µM	98.4 ± 0.8	*p* > 0.05
Dapagliflozin	1 µM	98.1 ± 1.3	*p* > 0.05
Canagliflozin	1 µM	98.5 ± 1.1	*p* > 0.05

## Data Availability

Data are contained within the article.
